# The effectiveness of herbal acupoint application for functional diarrhea: Protocol for a meta-analysis and data mining

**DOI:** 10.1097/MD.0000000000027702

**Published:** 2021-11-24

**Authors:** Baiyan Liu, Bing Yan, Hailin Jiang, Xuewei Zhao, Luyao Wang, Tie Li, Fuchun Wang

**Affiliations:** Changchun University of Chinese Medicine, No. 1035, Boshuo Rd, Jingyue Economic Development District, Changchun, China.

**Keywords:** complementary and alternative therapy, data mining, functional diarrhea, herbal acupoint application, protocol, systematic review

## Abstract

**Background:**

Functional diarrhea (FDr), one of the most common functional gastrointestinal diseases, is a kind of functional bowel disease characterized by repeated paste feces or watery feces. However, no relevant systematic review or meta-analysis has been designed to evaluate the effects of herbal acupoint application (HAA) on FDr. There is also a lack of systematic evaluation and analysis of acupoints and herbs.

**Methods:**

We will search the following 8 databases from their inception to October 15, 2021, without language restrictions: the Cochrane Central Register of Controlled Trials, PubMed, Embase, the Web of Science, the Chinese Biomedical Literature Database, the Chinese Scientific Journal Database, the Wan-Fang Database, and the China National Knowledge Infrastructure. The primary

outcome measures will be clinical effective rate, functional outcomes, and quality of life. Data that meets the inclusion criteria will be extracted and analyzed using RevMan V.5.3 software (Available at: https://community.cochrane.org/help/tools-and-software/revman-5). Two reviewers will evaluate the studies using the Cochrane Collaboration risk of bias tool. We will use the Grading of Recommendations Assessment, Development and Evaluation approach to assess the overall quality of evidence supporting the primary outcomes. We will also use SPASS software (Version 19.0 (Available at: https://www.ibm.com/analytics/spss-statistics-software)) for complex network analysis to explore the potential core prescription of acupoint herbal patching for FDr.

**Results:**

This study will analyze the clinical effective rate, bristol stool scale, number of daily bowel movements, clinical symptom scale of diarrhea, and effective prescriptions of HAA for patients with FDr.

**Conclusion:**

The conclusion of our findings will provide evidence for the effectiveness and potential treatment prescriptions of HAA for patients with FDr.

## 1 Introduction

Diarrhea is a major clinical manifestation of many gastrointestinal disorders, which is common reason for patient visit. When there is no secondary cause or Irritable Bowel Syndrome (IBS), chronic diarrhea is diagnosed as functional diarrhea (FDr). FDr, one of the most common functional gastrointestinal diseases, is a kind of functional bowel disease characterized by repeated paste feces or watery feces.^[1]^ FDr is a common problem, with incidence estimates at 5 per 100,000 per year, and prior infectious diarrhea is an important risk factor for it.^[2]^ Persistent FDr is common and associated with reduced quality of life,^[3,4]^ anxiety, and depression.^[5]^ Currently, management or treatment measures for FDr include dietary modification, drugs acting on the peripheral or the whole body, and gut microbiota and immune-regulatory agent, etc.^[1]^ Although these measures play an important role in the treatment of FDr, and relieve symptoms temporarily, these symptoms are always recurrent.^[6]^ Therefore, it is significant to seek new therapies for the treatment of FDr.

In recent years, traditional Chinese medicine has attracted extensive attention for its ability to treat gastrointestinal diseases due to its moderate treatment effect and lower side effect. Herbal acupoint application (HAA), a traditional Chinese medicine therapy suitable for some chronic diseases, has also been used to treat perennial allergic rhinitis, chronic obstructive pulmonary disease, insomnia, IBS-D.^[7–10]^ Clinical study has demonstrated that HAA plus conventional treatment has a better effect on ameliorating the symptoms than patients taking conventional treatment.^[11,12]^ Although many studies have shown that HAA is effective for patients with FDr. However, the clinical efficacy and potential treatment prescriptions of HAA for FDr remain unclear, prompting us to further explore its efficacy and effective prescription. In this study, we will investigate current evidence associated with the effectiveness and safety of acupuncture for FDr, which will help clinicians use it in clinical practice better.

## 2 Method

### 2.1 Study type

We will collect articles of randomized controlled trials which treat function diarrhea by HAA for systematic review and meta-analysis. Randomized controlled trials, observational studies, case series, and case-control studies will be included for data mining. Observational studies, case series, case-control studies, animal experiments, qualitative studies, proceedings, conferences, comments, and review articles will be excluded in the meta-analysis. There will be no restrictions regarding the race, region of the studies, gender, and age.

### 2.2 Participants

This review will include patients of any age who had been diagnosed with FDr without limitations related to gender, race, study area, and education status. The diagnosis of FDr needs to be consistent by either ROME II or III or IV, or any recognized diagnostic criterion.

### 2.3 Interventions

We will enroll articles that the intervention participants undergoing HAA, regardless of herbal formulation, acupoints selected, patching time. There are no restrictions on age, race/ethnicity, nationality, or other participant characteristics. Patients in the control group received medication, no treatment, sham or placebo acupoint catgut embedding, acupuncture/electro-acupuncture, and etc. All other interventions should be identical for intervention and control participants.

### 2.4 Outcome measures

The primary outcome measure was clinical effective rate, bristol stool scale, number of daily bowel movements, clinical symptom scale of diarrhea, while secondary outcome measures included number and types of adverse events.

### 2.5 Search strategy

An electronic search will be conducted. We will search the following 8 databases from their inception to October 15, 2021, without language restrictions: the Cochrane Central Register of Controlled Trials (CENTRAL), PubMed, EMBASE, the Web of Science, the Chinese Biomedical Literature Database (CBM), the Chinese Scientific Journal Database (VIP database), the Wan-Fang Database, and the China National Knowledge Infrastructure (CNKI). The search term will consist of 3 parts: intervention method, disease, and study type: (“acupoint application” or “ acupoint sticker” or “crude herb moxibustion” or “medicinal vesiculation” or “herbal patch” or “herbal plaster” or “acupoint patch” or “Sanfu” or “acupoint sticking” or“point application therapy” or“drug acupoint application” or “winter diseases treated with acupoint stimulation in summer” or “drugs and points for point application in summer to treat the diseases with attacks in winter” or “acupuncture point application therapies” or “plaster therapy” or “external application therapy” or “acupoint herbal patching”) and (“functional diarrhea” or “chronic functional diarrhea”) and (“randomized controlled trial” or “randomized” or “case control studies” or “observational studies” or “case series” or “trial”) and (“blind”). The details of the PubMed and Wan-Fang Database search strategies are provided in Tables 1 and 2. The similar but adaptive search strategies will be applied to other electronic databases. Language will be restricted to English and Chinese. Besides, we will screen the references of included studies to identify other potential clinical trials. We will also search the following 3 databases for prospectively registered and ongoing trials: the World Health Organization (WHO) International Clinical Trials Registry Platform (ICTRP) search portal (http://apps.who.int/trialsearch/Default.aspx); Clinical Trials.gov (http://ClinicalTrials.gov/); and Chinese Clinical Trials Registry (http://www.chictr.org.cn).

**Table 1:** The search strategy for PubMed database.

Number	Search terms
#1	acupoint appliaction [MeSH]
#2	acupoint sticker [MeSH]
#3	crude herb moxibustion [MeSH]
#4	medicinal vesiculation [MeSH]
#5	herbal patch [MeSH]
#6	acupoint patch [MeSH]
#7	Sanfu [MeSH]
#8	acupoint sticking [MeSH]
#9	point application therapy [MeSH]
#10	drug acupoint application [MeSH]
#11	winter diseases treated with acupoint stimulation in summer [MeSH]
#12	drugs and points for point application in summer to treat the diseases with attacks in winter [MeSH]
#13	acupuncture point application therapies [MeSH]
#14	plaster therapy [MeSH]
#15	external application therapy [MeSH]
#16	complementary and alternative medicine [MeSH]
#17	complementary and alternative therapy [MeSH]
#18	#1 or #2 or #3 or #4 or #5 or #6 or #7 or #8 or #9 or #10 or #11 or #12 or #13 or #14 or #15 or #16 or #17
#19	functional diarrhea [MeSH]
#20	chronic functional diarrhea [MeSH]
#21	#19 or #20
#22	randomized controlled trial [MeSH]
#23	case control studies [MeSH]
#24	observational studies [MeSH]
#25	case series [MeSH]
#26	trial [MeSH]
#27	#22 or #23 or #24 or #25 or #26
#28	#18 and #21 and #27

**Table 2:** The search strategy for Wanfang database.

Number	Search terms
#1	acupoint appliaction [MeSH]
#2	acupoint sticker [MeSH]
#3	crude herb moxibustion [MeSH]
#4	medicinal vesiculation [MeSH]
#5	herbal patch [MeSH]
#6	acupoint patch [MeSH]
#7	Sanfu [MeSH]
#8	acupoint sticking [MeSH]
#9	point application therapy [MeSH]
#10	drug acupoint application [MeSH]
#11	winter diseases treated with acupoint stimulation in summer [MeSH]
#12	drugs and points for point application in summer to treat the diseases with attacks in winter [MeSH]
#13	acupuncture point application therapies [MeSH]
#14	plaster therapy [MeSH]
#15	external application therapy [MeSH]
#16	#1 or #2 or #3 or #4 or #5 or #6 or #7 or #8 or #9 or #10 or #11 or #12 or #13 or #14 or #15
#17	functional constipation [MeSH]
#18	chronic functional constipation [MeSH]
#19	#17 or #18
#20	randomized controlled trial [MeSH]
#21	case control studies [MeSH]
#22	observational studies [MeSH]
#23	case series [MeSH]
#24	trial [MeSH]
#25	#20 or #21 or #22 or #23 or #24
#26	#16 and #19 and #25

### 2.6 Study selection and data extraction

**Figure 1:**
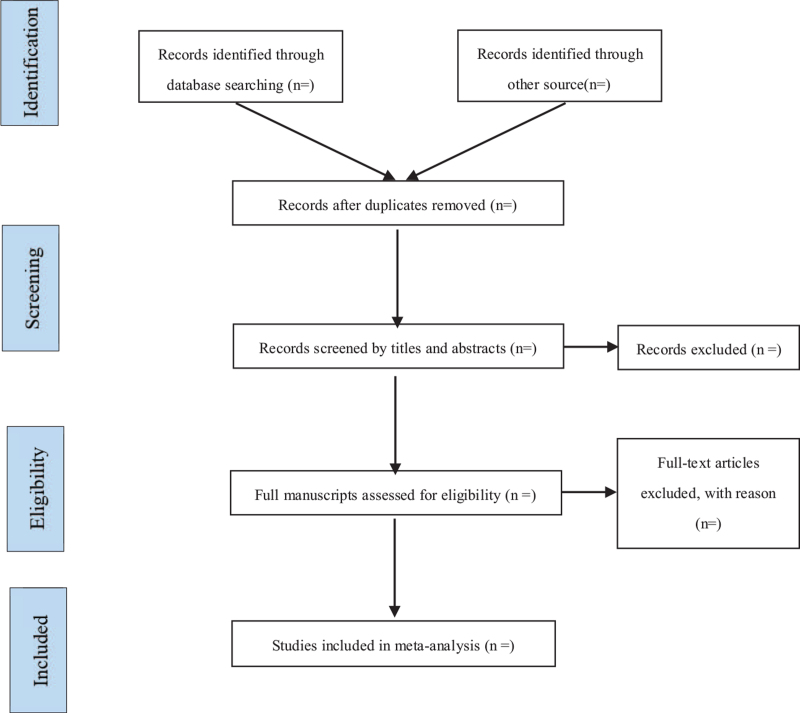
Flow chart of the search process.

Author (Zhao XW) with experience in the field will guide the search. First, the NoteExpress 3.2.0 (Available at: http://www.inoteexpress.com/aegean/) software will be used to exclude duplicate references from different databases. Two review authors (Liu BY and Yan B) will independently assess the title and abstracts of all citations found from the above search strategy. A copy of the full text article is obtained for the potentially eligible studies. These review authors will independently read the full text articles to include eligible studies; disagreement will be resolved by consensus through discussion with a third review author (Zhao XW). If conclusion still cannot be met, we will contact the author of the article to determine the eligibility of the study. The selection process will be showed in a PRISMA flow chart (http://www.prismastatementorg/) (Fig. 1). In the end, 2 review authors (Liu BY and Yan B) will extract data using a data extraction form according to the recommendations of the Cochrane Handbook for Systematic Reviews of Interventions. The following data will be extracted: author, year of publication, country where the study was conducted, study period, original inclusion criteria, total number of people included in the study, acupoints, doses of herbs and time of application, and etc.

### 2.7 Addressing missing data or unclear measurement scales

If the data were not provided or insufficient, we will contact the authors and for an attempt to get detailed information. Otherwise, we will analyze the available information and conduct sensitivity analysis to explore the potential impact of insufficient information on the results of the meta-analysis.

### 2.8 Risk of bias in included studies

Two review authors (Jiang HL and Liu BY) will independently evaluate each included study and will follow the domain-based evaluation as developed by the Cochrane Handbook for Systematic Reviews of Interventions. They will assess the following domains:

(1) selection bias (random sequence generation and allocation concealment),
(2) performance bias (blinding of participants and personnel),
(3) detection bias (blinding of outcome assessment),
(4) attrition bias (incomplete outcome data),
(5) reporting bias (selective reporting),
(6) other bias (such as pre-sample size estimation, early stop of trial).

Each domain will be divided into 3 categories: “low risk”,“high risk”, or “unclear risk”.

### 2.9 Data synthesis and analysis

We will analyze the data with RevMan software (Version 5.3) provided by the Cochrane Collaboration.^[13]^ A meta-analysis using random or fixed effects models will be conducted to summarize the data. Continuous data will be pooled and presented as mean differences or standardized mean difference with their 95% confidence interval. Dichotomous data will be pooled and expressed as risk ratio with their 95% confidence interval. We will interpret it using the following criteria: I^2^ values of 25% is considered low levels of heterogeneity, 50% indicated moderate levels, and 75% indicated high levels.^[14]^ Since low or moderate heterogeneity suggests little variability among these studies, the data will be analyzed in a fixed-effects model.^[15]^ When significant heterogeneity occurs among the studies (*P* < .05, I^2^ > 50%), a random-effect model will be performed to analyze the data.

### 2.10 Additional analyses

We will conduct the subgroup analysis to determine heterogeneity as per study information, intervention type, age, course of disease, treatment duration on pooled results. If the data are insufficient, qualitative synthesis will be conducted instead of quantitative synthesis.

The sensitivity inspection will be conducted to inspect the robustness of the study. We will eliminate low qualities articles one by one to inspect the reliability of this meta-analysis’ results. We will also use SPASS software (Version19.0) for complex network analysis to explore the potential core prescription of acupoint herbal patching for FDr.

### 2.11 Assessment of publication biases

Publication bias was evaluated by visually inspecting funnel plots. We performed Begg test and Egger test to assess publication bias (*P* < .05 was considered statistically significant).

### 2.12 Confidence in cumulative evidence

In order to better prepare results for usage in guideline development, we will assess the overall quality of evidence supporting the primary outcomes by the Grading of Recommendations Assessment, Development and Evaluation.^[16]^ Grading of Recommendations Assessment, Development and Evaluation will be used to summarize the limitations in design, consistency, directness, precision, publication bias. The quality of each evidence will be divided into 4 levels: high, medium, low, and very low. Disagreements will be resolved by consensus.

## 3 Discussion

Chronic, long-lasting functional diarrhea is a distressing condition, which has a negative impact on patients’ health, nutritional status, and quality of life. Previous studies have shown that, FDr prevalence ranges within1.5% to 17%.^[17–20]^ Notably, 94% of patients have diarrhea more than 12 months or over and nearly one-third lasted 10 years or more.^[6,21]^ The pathological mechanism of FDr is complex and multifactorial, including gastrointestinal dynamics, abnormalities in the brain-gut axis, previous infection, environmental and genetic factors, psychosocial, and other facts.^[22]^ Although various therapies for FDr are available, the therapeutic effects remain unsatisfactory. Therefore, many doctors and patients with FDr are actively exploring other complementary and alternative therapies for treatment. Although many studies have shown that HAA is effective for patients with FDr. No systematic review has been carried out to assess the efficacy and safety of HAA for FDr. Thus, this study will systematically evaluate the efficacy and safety of HAA for the treatment of patients with FDr. This study will assess the clinical efficacy and effective prescriptions of HAA for patients with FDr, and may benefit practitioners in the fields of complementary and alternative therapies.

## 4 Ethics and dissemination

The systematic reviews with no need for ethics approval because of not involved in individual data.

## Author contributions

**Conceptualization:** Baiyan liu, Bing Yan, Hailin Jiang.

**Data curation:** Xuewei Zhao, Luyao Wang.

**Formal analysis:** Baiyan liu, Bing Yan, Hailin Jiang.

**Funding acquisition:** Fuchun Wang.

**Investigation:** Bing Yan, Hailin Jiang.

**Methodology:** Tie Li.

**Project administration:** Tie Li, Fuchun Wang.

**Resources:** Fuchun Wang.

**Software:** Bing Yan, Hailin Jiang, Tie Li.

**Supervision:** Fuchun Wang.

**Validation:** Baiyan liu, Bing Yan.

**Visualization:** Fuchun Wang.

**Writing – original draft:** Baiyan liu.

**Writing – review & editing:** Hailin Jiang, Tie Li, Fuchun Wang.
